# A Case of Successful Transcatheter Coil Embolization of Multiple Systemic Arteries-to-Pulmonary Artery Fistulas

**DOI:** 10.7759/cureus.81327

**Published:** 2025-03-28

**Authors:** Hina Jojiki, Toshihiro Osaki, Shinji Shinohara, Soichi Oka, Hiroyuki Ueda

**Affiliations:** 1 Thoracic Surgery, Kokura Memorial Hospital, Kitakyushu, JPN; 2 Radiology, Kokura Memorial Hospital, Kitakyushu, JPN

**Keywords:** detachable coil, interventional radiology guided embolization, multiple systemic artery-to-pulmonary artery fistulas, transarterial coil embolization, transcatheter coil embolization

## Abstract

Pulmonary vascular malformations include pulmonary arteriovenous fistula, systemic artery-to-pulmonary artery fistula (SAPAF), and systemic artery-to-pulmonary vein fistula. In particular, systemic artery-to-pulmonary artery fistulas with three or more inflow vessels are rare. Herein, we report a case of successful transcatheter coil embolization for multiple SAPAFs. Three-dimensional computed tomography (3D-CT) and selective angiography revealed that the right inferior phrenic artery, right internal thoracic artery, and two right bronchial arteries were visualized and flowing into the right lower pulmonary artery through a 3 cm aneurysm. Under X-ray fluoroscopy guidance, a microcatheter was inserted into the aneurysm from the side of the main pulmonary artery, and the inflow arteries were embolized with coils. Subsequently, the interior of the aneurysm was embolized using coils. Follow-up angiography was performed six months postoperatively. Shunts between all systemic arteries and pulmonary arteries disappeared completely, and the small aneurysm was confirmed to have decreased in size. In this case, when a lung resection is performed, a large aneurysm is present, and vascular handling is expected to be challenging, so transcatheter arterial embolization is considered a minimally invasive and highly effective treatment.

## Introduction

Pulmonary vascular malformations include pulmonary arteriovenous fistula, systemic artery-to-pulmonary artery fistula (SAPAF), and systemic artery-to-pulmonary vein fistula. A SAPAF is a rare mention that often remains asymptomatic, with an uncertain clinical course, making definitive treatment indications unclear. A SAPAF can arise from either congenital or acquired causes and may alter the hemodynamics of the pulmonary circulation. Complications such as hemoptysis, hemothorax, massive bleeding from aneurysmal rupture, infections, right heart failure, and pulmonary hypertension have been reported [1], potentially justifying treatment even in asymptomatic patients.

Treatment options typically include surgical intervention and transcatheter therapy. Recently, transcatheter embolization has emerged as a viable treatment approach [2,3]. Here, we report a patient with SAPAF involving multiple feeding vessels who was treated with transcatheter coil embolization, resulting in the complete cessation of shunt flow.

## Case presentation

**Figure 1:**
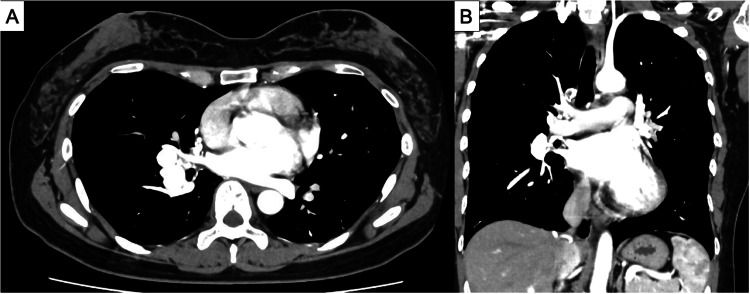
Preoperative contrast enhanced computed tomography (CECT) (A,B) An aneurysm is observed on the ventral side of the right lower pulmonary artery, and the aneurysm communicates with the right lower pulmonary artery.

A 40-year-old female patient was referred to our department with a diagnosis of SAPAFs, made incidentally during contrast-enhanced computed tomography (CECT) performed to evaluate a pancreatic cyst that was identified during a health checkup (Figures 1A, 1B). The patient’s medical history was unremarkable, except for one ectopic pregnancy and two cesarean sections. Physical examination revealed no remarkable findings, and blood tests and arterial blood gas analyses on room air were within normal. Echocardiography revealed no evidence of right heart failure or pulmonary hypertension.

**Figure 2:**
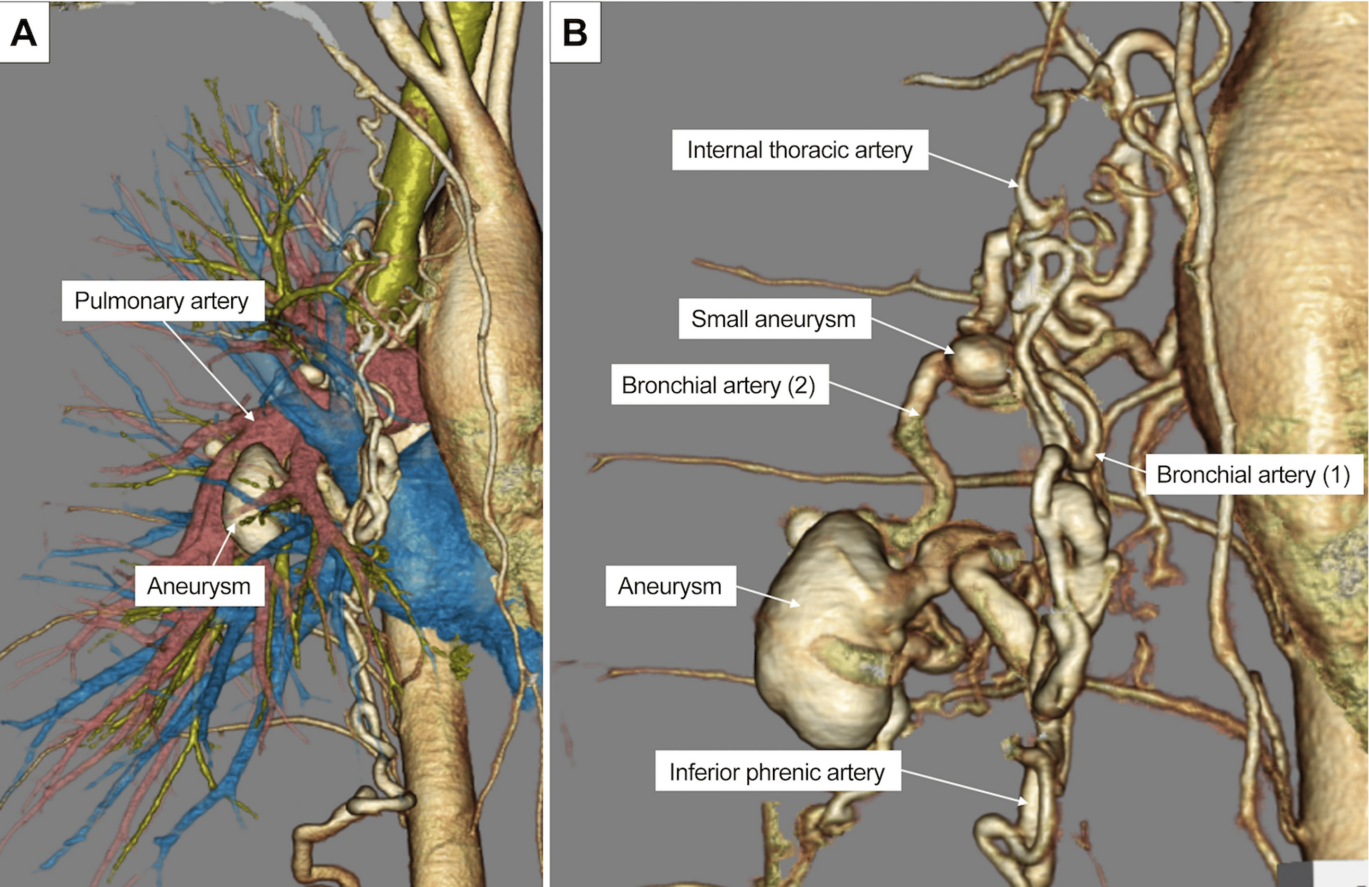
Three-dimensional computed tomography (CT) of the multiple systemic arteries-to-pulmonary artery fistula (A) Several abnormal systemic arteries flow into the right lower pulmonary artery after passing through a 3-cm aneurysm. (B) The right inferior phrenic, right internal thoracic, and two right bronchial arteries, along with a small aneurysm, are visualized, flowing into the aneurysm.

Three-dimensional CT (3D-CT) revealed several abnormal systemic arteries flowing into the right lower pulmonary artery through a 3-cm aneurysm (Figure 2A). Specifically, the right inferior phrenic artery, right internal thoracic artery, and two right bronchial arteries, as well as a small aneurysm, were visualized and connected to the right lower pulmonary artery through an aneurysm (Figure 2B).

**Figure 3:**
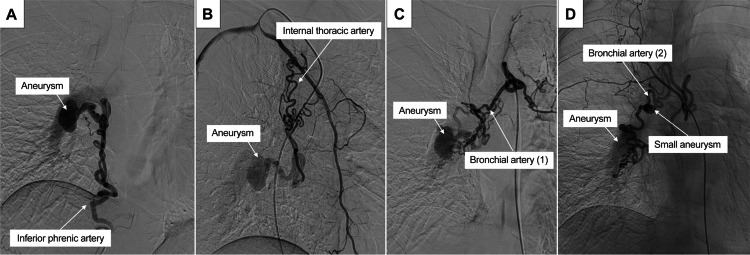
Selective angiography of the multiple systemic arteries-to-pulmonary artery fistula (A) The right inferior phrenic artery is seen flowing into the aneurysm and then into the pulmonary artery. (B) The right internal thoracic artery is visualized joining the inferior phrenic artery and then flowing into the aneurysm. (C) One of the bronchial arteries is seen joining the inferior phrenic artery and then flowing into the aneurysm. (D) Another bronchial artery with a small aneurysm is visualized flowing directly into the aneurysm.

Selective angiography revealed blood flow from the right inferior phrenic artery to the right lower pulmonary artery via the aneurysm (Figure 3A). The right internal thoracic artery joined the right inferior phrenic artery before flowing into the right lower pulmonary artery through the aneurysm (Figure 3B). The bronchial arteries showed two abnormal vessels: one merged with the right inferior phrenic artery, similar to the internal thoracic artery, before entering the aneurysm (Figure 3C), while the other, associated with a small aneurysm, flowed directly into the aneurysm (Figure 3D).

**Figure 4:**
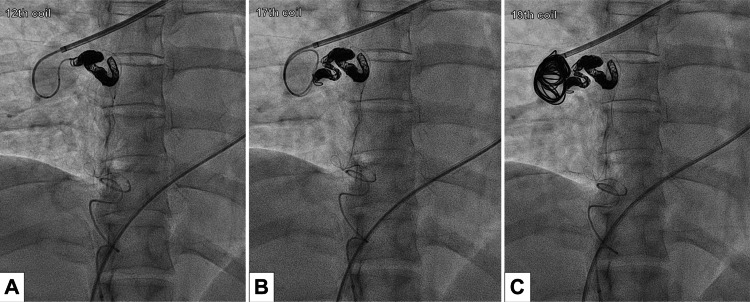
Transcatheter coil embolization from the pulmonary artery via the aneurysm (A) First, coil embolization was performed on the inflow artery where three arteries, the inferior phrenic artery, internal thoracic artery, and bronchial artery, converged. (B) Next, coil embolization was performed on the artery into which one bronchial artery directly flowed. (C) Finally, coil embolization was performed on the aneurysm itself.

During the transcatheter coil embolization, access was obtained via the right femoral artery and vein. A 3-Fr guiding sheath was advanced from the celiac artery to the right inferior phrenic artery, and an angiographic catheter was used to navigate the aneurysm. A 5-Fr guiding sheath was advanced into the right pulmonary artery from the venous side. Guided by contrast imaging from the right inferior phrenic artery, a microcatheter was advanced through the aneurysm into the two feeding arteries via the pulmonary artery. Both feeding arteries were embolized using detachable coils (Figures 4A, 4B). Subsequently, the interior of the aneurysm was embolized with coils (Figure 4C).

To further promote aneurysmal thrombosis, 33% n-butyl-2-cyanoacrylate was injected into the aneurysm. The shunts formed between the right inferior phrenic, right internal thoracic, right bronchial, and pulmonary arteries disappeared. As the bronchial small-artery aneurysm was expected to shrink due to reduced blood flow following the procedure, no additional treatment was performed. The patient was discharged without complications on postoperative day two.

**Figure 5:**
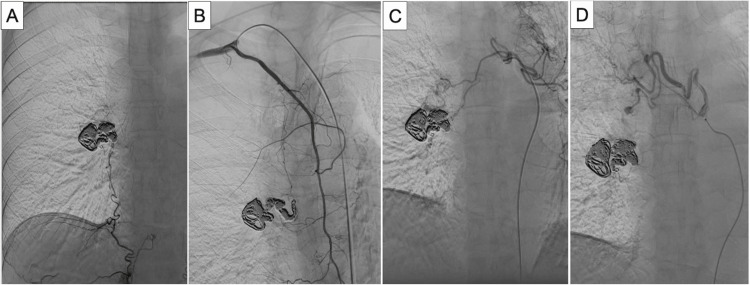
Selective angiography performed at six months postoperatively (A) The diameter of the inferior phrenic artery is reduced compared to the preoperative measurement, and the shunt flow to the pulmonary artery has disappeared. (B) The shunt flow from the right internal thoracic artery to the pulmonary artery has disappeared. (C) Angiography from the common trunk of the two bronchial arteries shows that the shunt to the pulmonary artery had disappeared. (D) The bronchial artery aneurysm remains but is smaller than in the preoperative state.

Follow-up selective angiography was performed six months postoperatively. Shunts between the right inferior phrenic, right internal thoracic, right bronchial, and pulmonary arteries disappeared completely (Figures 5A-5C). The small aneurysm observed during the surgery persisted but was confirmed to have decreased in size compared to that during the surgical state (Figure 5D). The patient remains asymptomatic, and the right bronchial artery aneurysm has thrombosed and further decreased in size.

## Discussion

A SAPAF can be congenital or acquired. Acquired SAPAF is often associated with chest trauma, malignant tumors, or infections, including nontuberculous mycobacterial infections and aspergillosis, although the underlying mechanisms remain unclear. Many patients with SAPAF are asymptomatic, and the condition is frequently diagnosed incidentally. In the current case, no acquired risk factors were identified, and the patient was asymptomatic.

Cases in which systemic and pulmonary arteries form shunts, as in this case, particularly those involving more than three inflow vessels, are rare [4,5]. Known feeding arteries include the bronchial, internal thoracic, inferior phrenic, and coronary arteries.

However, the optimal treatment method remains unknown. Recently, transcatheter coil embolization has been increasingly used because of its minimally invasive nature, preservation of lung parenchyma, and avoidance of general anesthesia [2,3]. In the present case, a right lower lobectomy or a combined middle and lower lobectomy was considered because of the aneurysm in the right lower pulmonary artery. However, the involvement of multiple systemic arteries connecting to the aneurysm in a tortuous manner made pulmonary vascular treatment extremely challenging.

In contrast, transcatheter treatment involves navigating the catheters through tortuous vessels and ensuring complete embolization of the feeding arteries to achieve effective flow cessation, which requires a high level of technical expertise. An accurate diagnosis and careful planning of treatment strategies are crucial for safe and effective management. Imaging modalities such as angiography and 3D-CT are valuable for understanding the vascular anatomy [5,6].

In transcatheter therapy, accessing the aneurysm from the systemic artery is challenging due to the tortuosity of the blood vessels, which complicates reliable catheter advancement. Conversely, pulmonary arterial access is technically challenging because it requires retrograde blood flow manipulation. In this case, we used a combined approach involving both systemic and pulmonary arterial accesses. Guided by contrast imaging of the right inferior phrenic artery, we navigated the pulmonary artery into the aneurysm and performed coil embolization of the feeding arteries.

## Conclusions

The choice between surgery and transcatheter treatment should be based on a comprehensive evaluation of technical expertise and the availability of the vascular anatomy using imaging studies. These findings should be carefully considered to estimate the degree of difficulty and associated risks before planning treatment. Furthermore, in cases like this one, a large aneurysm is present and vascular handling is expected to be challenging; transcatheter arterial embolization is considered a minimally invasive and highly effective.
